# Oceanography promotes self-recruitment in a planktonic larval disperser

**DOI:** 10.1038/srep34205

**Published:** 2016-09-30

**Authors:** Peter R. Teske, Jonathan Sandoval-Castillo, Erik van Sebille, Jonathan Waters, Luciano B. Beheregaray

**Affiliations:** 1 Molecular Ecology Lab, School of Biological Sciences, Flinders University, Adelaide, South Australia 5001, Australia; 2 Molecular Zoology Lab, Department of Zoology, University of Johannesburg, Auckland Park 2006, South Africa; 3 Grantham Institute & Department of Physics, Imperial College London, London SW7 2AZ, UK; 4 ARC Centre of Excellence for Climate System Science, University of New South Wales, Sydney, NSW 2052, Australia; 5 Department of Zoology, University of Otago, Dunedin 9054, New Zealand

## Abstract

The application of high-resolution genetic data has revealed that oceanographic connectivity in marine species with planktonic larvae can be surprisingly limited, even in the absence of major barriers to dispersal. Australia’s southern coast represents a particularly interesting system for studying planktonic larval dispersal, as the hydrodynamic regime of the wide continental shelf has potential to facilitate onshore retention of larvae. We used a seascape genetics approach (the joint analysis of genetic data and oceanographic connectivity simulations) to assess population genetic structure and self-recruitment in a broadcast-spawning marine gastropod that exists as a single meta-population throughout its temperate Australian range. Levels of self-recruitment were surprisingly high, and oceanographic connectivity simulations indicated that this was a result of low-velocity nearshore currents promoting the retention of planktonic larvae in the vicinity of natal sites. Even though the model applied here is comparatively simple and assumes that the dispersal of planktonic larvae is passive, we find that oceanography alone is sufficient to explain the high levels of genetic structure and self-recruitment. Our study contributes to growing evidence that sophisticated larval behaviour is not a prerequisite for larval retention in the nearshore region in planktonic-developing species.

Many coastal marine species have a two-phase life-cycle in which adults are sessile or sedentary, with dispersal instead facilitated by pelagic propagules such as eggs and planktonic larvae^1^,^2^. Given the small size of these ‘dispersive’ propagules, it has traditionally been assumed that they are transported passively by ocean currents^3^, and that the majority of larvae that settle in a particular area may have originated elsewhere in a species’ range^4^,^5^. Recent studies, however, have suggested that planktonic dispersal may not be as passive as previously assumed, but rather that propagule behaviour can promote larval retention in the vicinity of parental habitats^6^,^7^.

Direct measurements of propagule dispersal are almost invariably difficult to obtain^8^, so various indirect approaches may be required to help elucidate larval movement patterns. The application of high-resolution genetic data, such as polymorphic microsatellites, has confirmed that connectivity between populations of marine species with high theoretical dispersal potential is often lower than expected^9^,^10^,^11^,^12^. For example, while positive correlations among genetic and geographic distance have been viewed as a defining feature of low-dispersal species, such as those that lack a planktonic dispersal phase^13^,^14^, there is increasing evidence that geographic distance can also reduce connectivity in planktonic dispersers^11^,^15^,^16^,^17^.

In recent years, seascape genetics (i.e., the joint analysis of realized dispersal based on genetic data and potential larval dispersal based on advection connectivity simulations) has proven to be particularly powerful in helping to identify factors that limit connectivity in the oceans^11^,^18^,^19^. In the present study, we used a seascape genetic approach to determine how oceanography affects genetic connectivity in a widespread temperate Australian marine invertebrate. The study species, *Siphonaria diemenensis* (Quoy & Gaimard, 1833) is a common rocky shore limpet that occurs throughout southern and eastern temperate Australia^20^. It has a high fecundity^21^ and a planktonic larval dispersal phase^22^, and unlike many other coastal invertebrates, it is not genetically subdivided into regional genetic units whose ranges are linked to the region’s biogeographic provinces^23^,^24^. These features make *S. diemenensis* a particularly suitable model for studying the effect of ocean circulation on the connectivity of rocky shore fauna with theoretically high dispersal potential.

Although southern Australia is dominated by three ocean currents (the Leeuwin Current, the South Australian Current and the Zeehan Current), which together can potentially connect the region’s entire fauna over a distance > 5000 km^25^, realised dispersal is often surprisingly limited^10^,^11^. This paradox has sometimes been attributed to the fact that southern Australia’s offshore currents are relatively weak^10^. In contrast, recent seascape genetic analyses suggest that the lack of larval dispersal may be attributable to on-shelf circulation^11^. The continental shelf in some regions may be up to 100 km wide, and it is likely that most larvae never reach the shelf-edge boundary currents^11^. In the present study, we identify particularly high levels of self-recruitment, and demonstrate that near-shore oceanographic constraints play key roles in limiting the spread of passively-dispersing particles.

## Methods

### Sample collection

**Figure 1:**
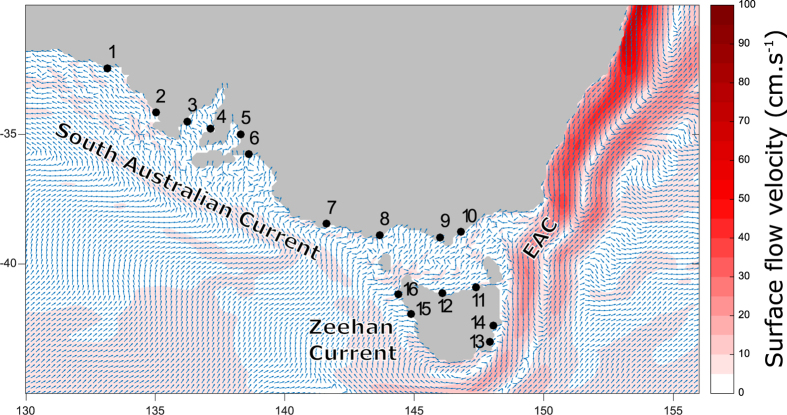
Study area. Sampling sites 1–16 correspond to those in Table S1. Arrows indicate the net direction of surface flow, and the colour gradient represents flow velocity, based on simulated advection from September 1 to November 30 (Model 2, see text). As a result of the wide continental shelf, many of the sampling sites are distant from major shelf-edge boundary currents, the South Australian Current, Zeehan Current and East Australian Current (EAC). The map was created with MATLAB 2015b (http://au.mathworks.com/).

Tissue samples from the foot of 714 individuals were collected from 16 localities (Fig. 1, Table S1) during 2011 and 2012, with sample sizes ranging from 29 to 48 (mean: 44.6 individuals). Samples from each locality generally included multiple size classes (e.g. shell length ranged from 5 to 28 mm). This strategy was used to avoid the inclusion of related individuals in the analysis and to include multiple cohorts, thus reducing issues associated with chaotic genetic patchiness due to stochastic recruitment^26^. Samples were stored in 99% ethanol, for no more than 1 month, until DNA was extracted using a salting-out protocol^27^. Thirteen microsatellites developed for *S. diemenensis* were amplified as described in^28^, namely *Side01, Side03, Side04, Side05, Side07, Side09, Side12, Side13, Side15, Side17, Side18, Side19* and *Side20*. Fragments were separated on an ABI 3730 Genetic Analyser (Applied Biosystems) and alleles were scored using GENEMAPPER v. 3.0 (Applied Biosystems). Tests for genotyping errors, null-alleles and large allele drop-out were performed with MICRO-CHECKER^29^, and tests for departures from Hardy-Weinberg equilibrium and linkage disequilibrum were done using Arlequin v. 3.5.2.1^30^. Arlequin was also used to calculate observed and expected heterozygosity at each site and for the complete data set, to identify the number of polymorphic loci and to report allele size ranges.

### Genetic structure and spatial analyses

To measure genetic structure between sites, we calculated pairwise *G*″_ST_ in GenAlEx 6.5^31^. This statistic is a nearly unbiased estimator of *G*′_ST_^32^ that should be used when sample sizes are small, making it suitable for pairwise comparisons^33^. For microsatellite data, it is preferable to the more commonly used *F*_ST_^34^ because it corrects for the maximum value possible when no alleles are shared between populations, which for microsatellites tends to be below the theoretical maximum of 1.0^33^. For comparison, we also calculated *F*_ST_. Significance of both statistics at α = 0.05 was assessed by running 999 permutations in GenAlEx, and the B-Y false discovery rate method^35^ was applied to account for multiple comparisons.

Geographic distances between sites were measured as minimum coastline distances in ARCMAP 10.1 (Environmental Systems Research Institute, Redlands, CA). GenAlEx was used to perform a paired Mantel test^36^,^37^ for a matrix comprising *G*″_ST_ values and one comprising geographic distances, and to construct a regression plot. Significance for the Mantel test was based on 999 permutations. GenAlEx was also used to perform a multilocus spatial autocorrelation analysis and to calculate within-population relatedness. Both methods are useful to determine whether larval recruitment occurs mostly in close proximity to the parent site, in which case the spatial autocorrelation coefficient *r*^38^ is expected to be greater than expected under a null hypothesis of no spatial genetic structure at smaller distance classes. This is because individuals that were collected in close geographic proximity to each other are expected to be more closely related to each other than they are to individuals from more distant sites. Statistical significance was based on 999 permutations to estimate 95% confidence intervals around the null hypothesis, and 1000 bootstrap replications to estimate the 95% confidence interval around *r*. Spatial genetic structure is present when the value of *r* is beyond the confidence interval of the null hypothesis and when the confidence interval of *r* does not overlap with zero. After exploring a number of distance class sizes, we presented an autocorrelation correlogram with a distance class size of 120 km, as this resulted in particularly good resolution for our sampling design.

As an independent and complimentary means of assessing levels of self-recruitment, relatedness or kinship indices have proven to be useful because individuals from the same site are more likely to be closely related to each other than they are to individuals from other sites^39^. We calculated within-population relatedness using the relatedness coefficient *r*^40^ for pairs of individuals from the same site (referred to here as *r*_QG_ to distinguish it from the spatial autocorrelation statistic). Genotypes from all sites were permuted 999 times to calculate 95% confidence intervals for the range of *r*_QG_ expected under conditions of spatial genetic homogeneity.

### Relationships between empirical genetic data and oceanography

The effect of ocean circulation on genetic connectivity was assessed using oceanographic connectivity simulations. These were performed on OFES hydrodynamic data^41^ at 5 m depth with the Connectivity Modeling System^42^ using two models that take into consideration information on the species’ spawning period^43^. The first (Model 1) assumed a short peak spawning season (October 1–31) and negligible recruitment for other months, while the second (Model 2) assumed that spawning occurred over a period of 3 months (September 1 to November 30). In both cases, particles were released every hour from each of the 16 sites for the years 1980 to 2009, and were advected for 30 days. Unlike in the closely related *S. denticulata*, where there is a lag of 2–3 months between hatching and settlement^44^, no lag was observed in S*. diemenensis*^43^, suggesting that this species’ planktonic larval duration is shorter and 1 month larval duration adequate. For Model 1, 15 particles were released every hour, and 5 particles were released every hour for Model 2 (as Model 2 has a three times longer spawning season), resulting a release of ~5.3 million particles for both models. We further differentiated between 1 spawning cycle (hereafter referred to as ‘1 generation’, given a generation time of 1 year) and 5 spawning cycles (hereafter ‘5 generations’) in which a particular individual could take part. For the model that included 5 spawning cycles, following the first spawning event the number of both locally retained and imported particles was determined for each site, and used to determine the particles released from that locality for the subsequent spawning cycle. These patterns of particle dispersal were averaged across the 30 years (i.e. from 1980 to 2009). See^11^ for additional details on the oceanographic connectivity simulations.

Relationships between *G*″_ST_ values and oceanographic connectivity matrices was assessed using Mantel tests, as described earlier, and Multiple Regression on Distance Matrices (MRDM^45^,^46^). The Mantel tests were performed between the matrix of *G*″_ST_ values and one of four oceanographic connectivity matrices (Model 1 for 1 generation; Model 1 for 5 generations; Model 2 for 1 generation; and Model 2 for 5 generations). As the value of *G*″_ST_ for each pair of sites is a consequence of both immigration and emigration, a single oceanographic connectivity value was calculated for each pair of sites as the sum of the number of settlers released from a particular site that reached the other site, and the number of settlers received from that site. In addition, as the inferred number of settlers differed by several orders of magnitude, each estimate was corrected for the Mantel test by taking its natural logarithm.

As Mantel tests have been criticised for having an inflated Type I error rate^47^, MRDM was used as an alternative approach to corroborate the results from the Mantel tests. MRDM is a multivariate method that uses multiple regression to simultaneously test for correlations between a dependent variable and one or more explanatory variables. Our ln-transformed explanatory variables had high levels of collinearity, with VIF (Variance Inflation Factors) for 3 out of 4 advection connectivity models being >5.0 (range: 3.9 to 10.5) when all models were analysed together (5 is considered to be the maximum acceptable VIF^48^). Because of this, we analysed data sets with only 1 or 2 explanatory variables. In the latter cases, one of the four advection connectivity models was simultaneously analysed with geographic distance, as VIF values were lower for these combinations (range: 1.7 – 2.7), with the exception of the combination that included Model 1 with 5 generations, which had a much higher VIF value (6.9) and was excluded. MRDM analyses were run in the R package ECODIST^49^, and significance was based on 10 000 permutations.

To determine how well the simulated oceanographic data explained the self-recruitment inferred with the genetic data, Spearman rank correlations^50^ in SigmaStat 1.0 (Systat Software, San Jose, CA) were used to assess whether mean relatedness at specific sites was correlated with the number of particles that returned to the release site. We also used this test to assess how particles reaching the continental shelf (i.e. water with a depth of >100 m) affected self-recruitment and larval loss.

## Results

All microsatellite loci were variable in samples from each site. The number of alleles per locus ranged from 5 (locus *Side*18) to 31 (*Side*20) for the combined data, and for individual sampled sites, it ranged from 2 (*Side*18 at sites 2 and 13) to 23 (*Side*09 at site 5). The mean number of alleles across all loci was similar at the different sampled sites and ranged from 11.2 to 12.7 (Table S1). Observed and expected heterozygosity were high (Table S1), with an average of 0.79 and 0.81, respectively. There was no evidence for null alleles, sequencing errors or large allele dropout. All loci were thus considered suitable for inclusion in population genetic analyses.

Of the *G*″_ST_ values calculated for 120 pairs of sites (Table 1), 85 were significant after correction for multiple tests (P < 0.015: 22 comparisons, P < 0.003: 63 comparisons). Uncorrected *F*_ST_ values were lower (Table S2), but the number of pairwise comparisons that were significant was very similar (P < 0.015: 18 comparisons, P < 0.003: 66 comparisons).

**Figure 2:**
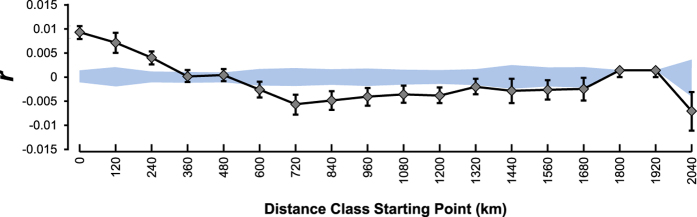
Correlogram of distance classes against the autocorrelation statistic *r* in the limpet *Siphonaria diemenensis* from southern Australia. The shaded blue area represent 95% confidence intervals under the condition of genetic homogeneity, and whiskers are 95% confidence intervals of *r*. Significant departures from the assumption of panmixia are evident when *r* is greater than the upper bound of the range indicated by the shaded blue area, and when its 95% confidence interval is greater than zero.

A Mantel test performed on a matrix of pairwise geographic distances and a matrix of pairwise values of *G*″_ST_ was highly significant (*R*xy = 0.385, P = 0.002), indicating that genetic structure can be described by a pattern of isolation by geographic distance. This was also evident in the positive correlation identified in the corresponding regression plot (Fig. S1). A spatial autocorrelation correlogram revealed that this relationship between genetic and geographic distances was primarily due to self-recruitment, with individuals from the same site being particularly closely related to each other (Fig. 2). The autocorrelation parameter *r* then decreased, and from 360 km onwards, there was no longer any clear trend in spatial patterns.

**Figure 3:**
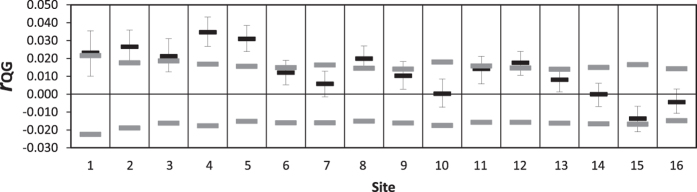
Mean pairwise relatedness within sites in the limpet *Siphonaria diemenensis* from southern Australia. Plots depict the relatedness coefficient *r*_QG_ (black bars) among individuals sampled at each of the 16 localities. Grey bars represent 95% confidence intervals expected under conditions of genetic homogeneity, and whiskers are 95% confidence intervals about *r*_QG_. Significant self-recruitment is evident when *r*_QG_ is greater than the upper bound of the range indicated by the grey bars, and when its 95% confidence interval is greater than zero.

Mean pairwise relatedness within sites (Fig. 3) confirmed that the highly significant positive spatial autocorrelation at the lowest distance class size (Fig. 2) was explained by high levels of self-recruitment, which was evident at seven sites. The relatedness coefficient *r*_QG_ was significantly greater than expected under conditions of panmixia at the five westernmost sites (and particularly high at the two sites located within the South Australian gulfs), but also at sites 8 and 12.

**Figure 4:**
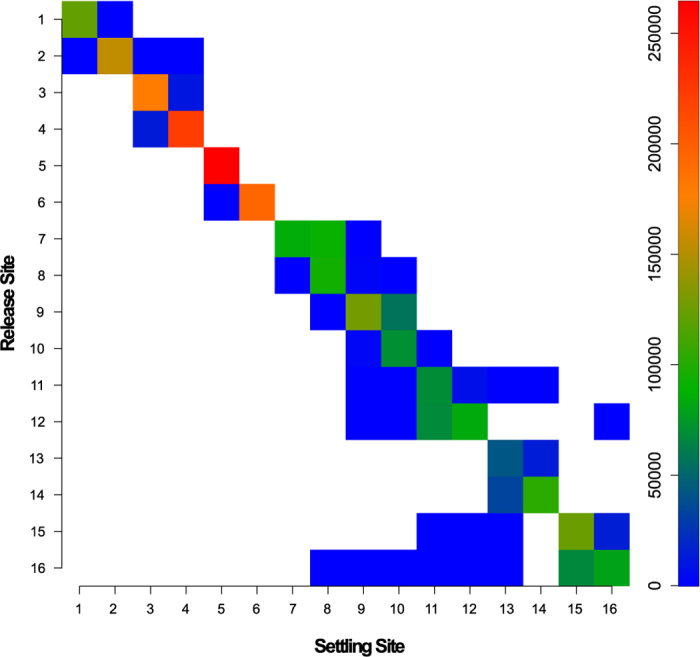
Simulated migration between pairs of sites. The present figure for migration rates of the planktonic larvae of the limpet *Siphonaria diemenensis* was based on ‘long’ oceanographic connectivity simulations (Model 2 for 1 generation). The diagonal represent self-recruitment. Please see Fig. S3 for the corresponding figure for Model 1, 1 generation.

Oceanographic connectivity simulations indicated that the high levels of self-recruitment shown in Figs 2 and 3 were largely a result of larval retention in the nearshore area (Figs 1 and 4, Fig. S2; Supplementary Materials: Animation). The net direction of surface flow was in most cases shoreward, making it very unlikely that advected particles would reach the region’s boundary currents, with eastern Tasmania being the only clear exception (Fig. 1). As a result, most advected particles settled at the release site, and recruitment beyond neighbouring sites was limited. This trend was particularly strong in the western portion of the species’ range, where high levels of self-recruitment were inferred for sites within the South Australian gulfs (sites 4 and 5), but also at more exposed sites (3 and 6). Mantel tests on matrices of ln-transformed connectivity simulated for a single generation against the genetic structure statistic *G*″_ST_ revealed a significant negative correlation for both the short spawning model, Model 1 (*R*xy = −0.50, P = 0.006) and the long spawning model, Model 2 (*R*xy = −0.53, P = 0.003). For the 5 generation simulations, the relationship was non-significant for Model 1 (*R*xy = −0.34, P = 0.07) but remained significant for the more realistic Model 2 (*R*xy = −0.477, P = 0.005). The two matrices have an inverse relationship because the more strongly two sites are connected by migration, the lower *G*″_ST_ is for that pair of sites (Fig. S3).

The results of the MRDM analyses were congruent with those of the Mantel tests. Tests were significant for all single explanatory variables, with the exception of Model 1, 5 generations (C in Table S3). On the basis of *F*-tests, the best model was Model 2, 1 generation (D in Table S3). Analyses that included more than one explanatory variable were all significant, but *F*-statistics were intermediate.

Mean relatedness (Table S4) at each of the 16 sites was positively correlated with simulated self-recruitment (*R* = 0.56, P = 0.02). A positive correlation was also found between the number of simulated particles that reached the shelf edge (Table S3) and those that failed to return to the coast to settle (*R* = 0.64, P < 0.01), and there was a negative correlation between those particles that reached the continental shelf and the level of self-recruitment (*R* = −0.71, P < 0.01). This indicates that particles that became entrained in the region’s shelf-edge boundary currents were not only less likely to return to the parent habitat, but also had lower recruitment success.

## Discussion

Evaluating how planktonic larvae recruit back into their source populations, and how much gene flow takes place between different populations, is not only critical for understanding the population dynamics of coastal species, but also to manage and conserve such populations^1^,^51^. In coastal populations, which tend to be characterized by weak signals of population differentiation, a pattern of isolation by geographic distance is often the only indication for a departure from the assumption of panmixia^51^. As discussed below, the joint analysis of genetic and oceanographic information represents a powerful means of elucidating factors underlying limited dispersal.

In the present study, a simple advection connectivity model, which treated larvae as passive particles and assumed that they remained in the surface layer throughout their dispersal phase, was adequate to explain high levels of genetic structure and self-recruitment. The considerable differences in these estimates at specific sites in the sampling region are readily explained by differences in how oceanographic regimes influence larval dispersal. Self-recruitment was particularly high in the west of the sampled range, as indicated by the high proportion of simulated particles that settled at their natal site and the high levels of relatedness among individuals from almost half of the study sites. In contrast, mean relatedness was low at most eastern sites, where the larger number of simulated particles reaching the continental shelf was positively correlated with greater larval loss. This difference between regions is not simply due to the presence of large bays in the west, where larval retention can be expected to be high^52^, as several of the more exposed sites also had circulation patterns that promoted self-recruitment. These results support the idea that in areas of South Australia where the continental shelf is particularly wide, planktonic propagules are primarily affected by local wind-driven circulation and coastal trapped waves^53^,^54^. The findings of the seascape genetic approach employed here are consistent with results of previous modelling work for a teleost fish, which suggested that recruitment in South Australia occurs at a highly localized scale, even when larval duration exceeds 100 days^55^. In contrast, at sites where the continental shelf is narrow and larvae are more likely to be advected by high velocity currents (e.g. in eastern and western Tasmania), self-recruitment is considerably less important. Studies conducted elsewhere confirm that a wide continental shelf can significantly reduce the influence of high-velocity offshore currents^56^,^57^ whereas long-distance dispersal by means of shelf-edge currents is more likely when the continental shelf is narrow^58^. In species that actively migrate to specific areas to spawn, the spawning areas are typically located where the shelf is particularly wide, which minimizes larval loss^56^. However, shelf width *per se* does not necessarily determine high levels of self-recruitment. Depending on local conditions, larval concentrations may decrease significantly from the coast to the shelf edge^59^, or on-shelf circulation may at least temporarily facilitate dispersal over the entire continental shelf 57.

The strong correlation between genetic structure and oceanography identified in this study is likely to apply more widely to most southern Australia’s mollusks that disperse by means of planktotrophic veliger larvae. Unlike many fish and crustaceans, whose late-stage larval often have considerable swimming ability and sensory competency that may reduce the influence of ocean currents on their dispersal^60^,^61^,^62^, veligers have comparatively low swimming abilities, and it is often assumed that they disperse like passive particles over large distances^63^,^64^,^65^. In addition, even though settlement behaviour can be quite sophisticated^66^, veliger behaviour during the dispersal phase tends to be limited to “reverse diel migration” (rising to the surface at night and sinking during daytime), which serves to avoid predators and exposure to ultraviolet radiation^67^. This is very different from the advection avoidance achieved in some fish larvae through a benthic lifestyle^68^. For that reason, veliger behaviour is less likely to promote nearshore retention in the presence of strong surface flows, which may explain why the dispersal of veliger larvae can be accurately predicted exclusively on the basis of hydrographic data^65^. Although a model that assumes passive dispersal of veligers is clearly a simplification, and a more sophisticated model may result in stronger correlations between connectivity simulations and genetic data, such a model is unlikely to challenge the finding of the present study that self-recruitment is driven by the hydrodynamic regime of the south Australian continental shelf.

In addition to assuming passive dispersal, other simplifications may have affected our simulations. First, we simulated larval dispersal from early September to the end of November^43^, yet *S. diemenensis* seems to be quite flexible in terms of when it spawns. Recruitment has also been reported between May and July^69^, when current flow along the continental shelf is strongest, and continuous eastward shelf-edge flow develops that can theoretically disperse planktonic larvae from the Australian south-west coast all the way to Tasmania^25^,^70^. The fact that this current flow occurs far offshore, while most larvae remain in the nearshore region where its influence is much diminished^71^, suggests that its importance in connecting sites throughout the region is insignificant in areas where the continental shelf is wide.

Secondly, our simulations may also have been affected by the fact that there is no detailed information on the planktonic larval duration of *S. diemenensis*. While larval duration can be inferred indirectly on the basis of spawning and recruitment patterns^43^, it is possible that the 1-month larval duration used here is overly conservative. The eggs of *S. diemenensis*^22^ are only slightly smaller than those of the often co-distributed *S. denticulata*, whose larvae also hatch well developed and may remain pelagic for up to 10 weeks^44^. If one assumes that egg size is positively correlated with developmental stage at hatching, because congeners that hatch fully developed have the largest eggs^21^, then a ~2-month larval duration is feasible for *S. diemenensis*. This is also unlikely to significantly affect genetic structure because there is no clear difference in realised dispersal distances of species whose larvae are part of the plankton for a few days compared to those that remain pelagic for weeks^72^,^73^.

## Conclusion

Our study contributes to the growing evidence that nearshore hydrodynamic processes can promote the retention of larvae close to parental habitats, even in ‘dispersive’ taxa^11^,^74^,^75^,^76^. On the wide continental shelf of South Australia, where the influence of the region’s shelf-edge boundary currents is minimal, complex larval behaviour is not necessary to explain high levels of self-recruitment. These findings have important implications for the design of marine reserves. The long-term persistence of populations within reserves is facilitated by a combination of self-recruitment and connectivity with other populations^77^. Our study supports the idea that, in the case of South Australian rocky shore fauna, an approach that relies on closely spaced networks of small reserves^78^ represents a more suitable management approach than the design of large but geographically distant reserves, because the majority of larvae will not disperse over greater distances^55^.

## Additional Information

**How to cite this article**: Teske, P. R. *et al*. Oceanography promotes self-recruitment in a planktonic larval disperser. *Sci. Rep.* **6**, 34205; doi: 10.1038/srep34205 (2016).

## Supplementary Material

Supplementary Information

Supplementary Animation

## Figures and Tables

**Table 1:** *G*″_ST_ values for pairs of sites in the limpet *Siphonaria diemenensis* from southern Australia.

	1	2	3	4	5	6	7	8	9	10	11	12	13	14	15
1															
2	0.05**														
3	0.01	0.03													
4	0.02	0.08**	0.05**												
5	0.03*	0.07**	0.05**	0.01											
6	0.04**	0.05**	0.05**	0.01	0.01										
7	0.04*	0.03	0.03*	0.08**	0.06**	0.06**									
8	0.04*	0.05**	0.05**	0.07**	0.06**	0.07**	0.02								
9	0.04*	0.04**	0.02	0.08**	0.10**	0.07**	0.02	0.02*							
10	0.03	0.04**	0.05**	0.09**	0.08**	0.07**	0.01	0.02	0.02						
11	0.05*	0.05**	0.03*	0.05**	0.06**	0.04**	0.02	0.02	0.01	0.00					
12	0.05*	0.09**	0.05**	0.05**	0.05**	0.03**	0.03*	0.03*	0.02	0.05**	0.01				
13	0.04**	0.05**	0.03*	0.06**	0.07**	0.06**	0.02	0.02*	0.01	0.03*	0.02	0.03*			
14	0.05**	0.04**	0.02	0.05**	0.04**	0.03**	0.02	0.06**	0.03*	0.03*	0.00	0.02*	0.01		
15	0.06**	0.04**	0.03	0.05**	0.05**	0.03**	0.01	0.06**	0.03*	0.03**	0.02	0.01	0.04**	0.000	
16	0.05**	0.05**	0.04*	0.06**	0.06**	0.03*	0.03*	0.06**	0.05**	0.04**	0.02	0.04**	0.05**	0.007	0.009

Significance following correction for multiple tests is indicated as ^*^α = 0.05 (corrected P-value: 0.015) and ^**^α = 0.01 (corrected P-value: 0.003). Site numbers correspond to those in Table S1.
