# Supplementary material for: Oceanography promotes self-recruitment in a planktonic larval disperser
Source: Sci Rep. 2016 Sep 30;6:34205. doi: 10.1038/srep34205 (PMC5043232; doi:10.1038/srep34205)
Supplement: Supplementary Information [file srep34205-s1.pdf]

Oceanography promotes self-recruitment in a planktonic larval disperser

Peter R. Teske[1,2], Jonathan Sandoval-Castillo[1], Erik van Sebille[3,4], Jonathan Waters[5] and Luciano B. Beheregaray[1,*]

[1]Molecular Ecology Lab, School of Biological Sciences, Flinders University, Adelaide, South Australia 5001, Australia; [2]Molecular Zoology Lab, Department of Zoology, University of Johannesburg, Auckland Park 2006, South Africa; [3]Grantham Institute & Department of Physics, Imperial College London, London SW7 2AZ, UK; [4]ARC Centre of Excellence for Climate System Science, University of New South Wales, Sydney, NSW 2052, Australia; [5]Department of Zoology, University of Otago, Dunedin 9054, New Zealand.

*Correspondence and requests for materials should be addressed to L. B. B. (email: luciano.beheregaray@flinders.edu.au)

Supplementary Material

Supplementary Tables

Table S1 | Names and locations of sampling sites, number of individuals per site for which microsatellite data were generated, observed (H_o) and expected (H_e) heterozygosity, and the mean number of alleles at each site.

Site No.	Site Name	GPS coordinates	No. samples	H_o	H_e	Mean no. alleles
1	Penong	32°05'14"S, 132°58'51"E	29	0.803	0.801	10.7
2	Point Drummond	34°08'30"S, 135°14'49"E	39	0.754	0.796	11.2
3	Peak Bay	34°29'22"S, 136°04'39"E	39	0.803	0.799	11.8
4	Point Souttar	34°53'44"S, 137°14'59"E	45	0.789	0.799	11.2
5	Glenelg	34°58'50"S, 138°30'35"E	47	0.789	0.794	12.3
6	Victor Harbour	35°33'01"S, 138°37'34"E	48	0.779	0.795	11.8
7	Portland	38°20'13"S, 141°36'34"E	47	0.805	0.808	12.2
8	Marengo	38°46'50"S, 143°39'59"E	48	0.762	0.802	12.0
9	Walkerville	38°51'39"S, 146°00'14"E	48	0.790	0.807	12.6
10	Bridport	40°58'54"S, 147°23'08"E	40	0.787	0.812	12.0
11	Penguin	41° 6'48"S, 146° 4'33"E	46	0.753	0.803	12.1
12	Port Albert	38°40'04"S, 146°41'60"E	48	0.760	0.801	11.5
13	Pirates Bay	43°00'33"S, 147°55'58"E	48	0.790	0.807	12.3
14	Swansea	42°07'12"S, 148°04'28"E	48	0.799	0.816	12.2
15	Trial Harbour	41°55'52"S, 145°10'18"E	46	0.816	0.824	12.7
16	Couta Rocks	41°10'29"S, 144°40'53"E	48	0.798	0.818	11.5
		Total:	714			

Table S2 | F_{ST} values for pairs of sites in the limpet *Siphonaria diemenensis* from southern Australia. Significance following correction for multiple tests is indicated as *α= 0.05 (corrected P-value: 0.015) and **α=0.01 (corrected P-value: 0.003). Site numbers correspond to those in Table S1.

	1	2	3	4	5	6	7	8	9	10	11	12	13	14	15
2	0.013**														
3	0.009	0.009													
4	0.010	0.015**	0.011**												
5	0.010	0.013**	0.011**	0.007											
6	0.011**	0.011**	0.011**	0.006	0.006										
7	0.011**	0.009	0.009*	0.013**	0.011**	0.011**									
8	0.011*	0.011**	0.011	0.012**	0.012**	0.012**	0.007								
9	0.011*	0.010**	0.007**	0.014**	0.015**	0.012**	0.008	0.008*							
10	0.012**	0.012**	0.010**	0.011**	0.012**	0.010**	0.008	0.008	0.007						
11	0.012**	0.015**	0.011**	0.011**	0.010**	0.009**	0.008*	0.009**	0.008	0.007					
12	0.010*	0.011**	0.011**	0.015**	0.014**	0.012**	0.006	0.008	0.007	0.006	0.010**				
13	0.013**	0.010**	0.008*	0.011**	0.010**	0.008*	0.006	0.011**	0.008*	0.008	0.007	0.009**			
14	0.012**	0.011**	0.010**	0.011**	0.011**	0.008*	0.008*	0.011**	0.010**	0.008	0.009**	0.009**	0.006		
15	0.012**	0.010**	0.008	0.011**	0.009**	0.008*	0.007	0.011**	0.008*	0.006	0.008	0.008*	0.005	0.006	
16	0.011*	0.011**	0.009*	0.011**	0.012**	0.011**	0.007	0.008	0.006	0.008	0.008*	0.008*	0.009**	0.010**	0.006

Table S3 | MRDM results for regressions between G''_{ST} as dependent variable and various combinations of explanatory variables; A: geographic distance; B-E: advection connectivity models.

Explanatory variables	F	R^2	P	Coef	P
A	544	0.0570	0.0001	9.9×10^{-6}	0.0001
B	650	0.0661	0.0001	5.0×10^{-4}	0.0001
C	2	0.0002	0.7082	2.1×10^{-6}	0.7082
D	703	0.0711	0.0001	5.3×10^{-4}	0.0001
E	234	0.0249	0.0001	1.9×10^{-4}	0.0001
A + B	474	0.0936	0.0001	A: 7.3×10^{-6}	A: 0.0001
				B: 4.0×10^{-4}	B: 0.0001
A + C	418	0.0836	0.0001	A: 1.5×10^{-5}	A: 0.0001
				C: -2.9×10^{-4}	B: 0.0003
A + D	475	0.0937	0.0001	A: 6.7×10^{-6}	A: 0.0003
				D: 4.2×10^{-4}	B: 0.0001

Explanatory variables: A: geographic distance; B: Model 1, 1 generation; C: Model 1, 5 generations; D: Model 2, 1 generation; Model 2, 5 generations. Model A + E was not used because of high levels of collinearity (VIF = 6.9); F = F-test statistic for lack of fit; R^2 = regression R^2 from the permutation test; Coef = regression coefficient from the permutation test.

Table S4 | The fate of 327,600 simulated particles that were released from each of 16 coastal sites in south-eastern Australia.

Site No.	Self-recruitment	No settlement	Shelf-edge is reached
1	122529	204983	2641
2	154743	172821	49047
3	179489	137034	19
4	221708	92327	69
5	264321	63279	3528
6	193624	133970	385
7	85529	151105	72932
8	94250	230238	4930
9	128190	142436	11266
10	70515	253708	74934
11	69608	250314	159927
12	83533	175333	260478
13	42329	270528	327254
14	103522	189799	309366
15	124917	187096	235958
16	80244	178756	119340

Supplementary Figures

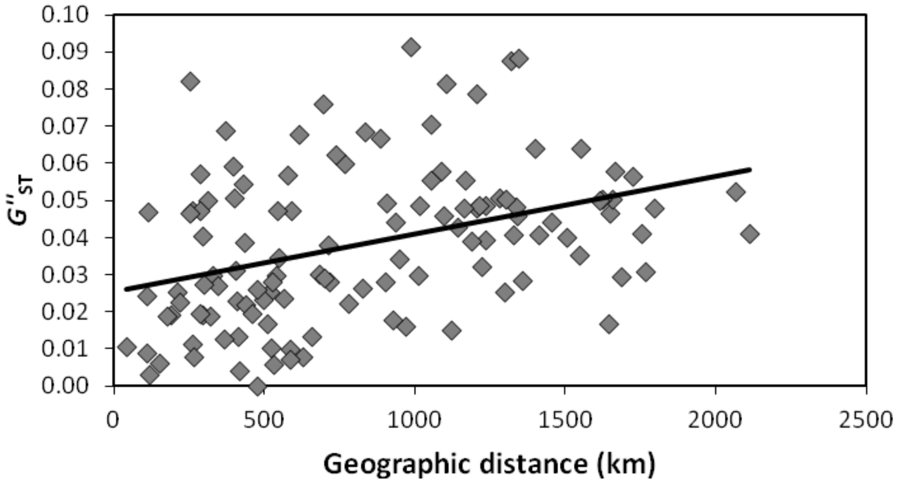

Figure S1. Regression plot for geographic distance against the genetic structure statistic. Shortest along-coast distances were plotted against G''_{ST}.

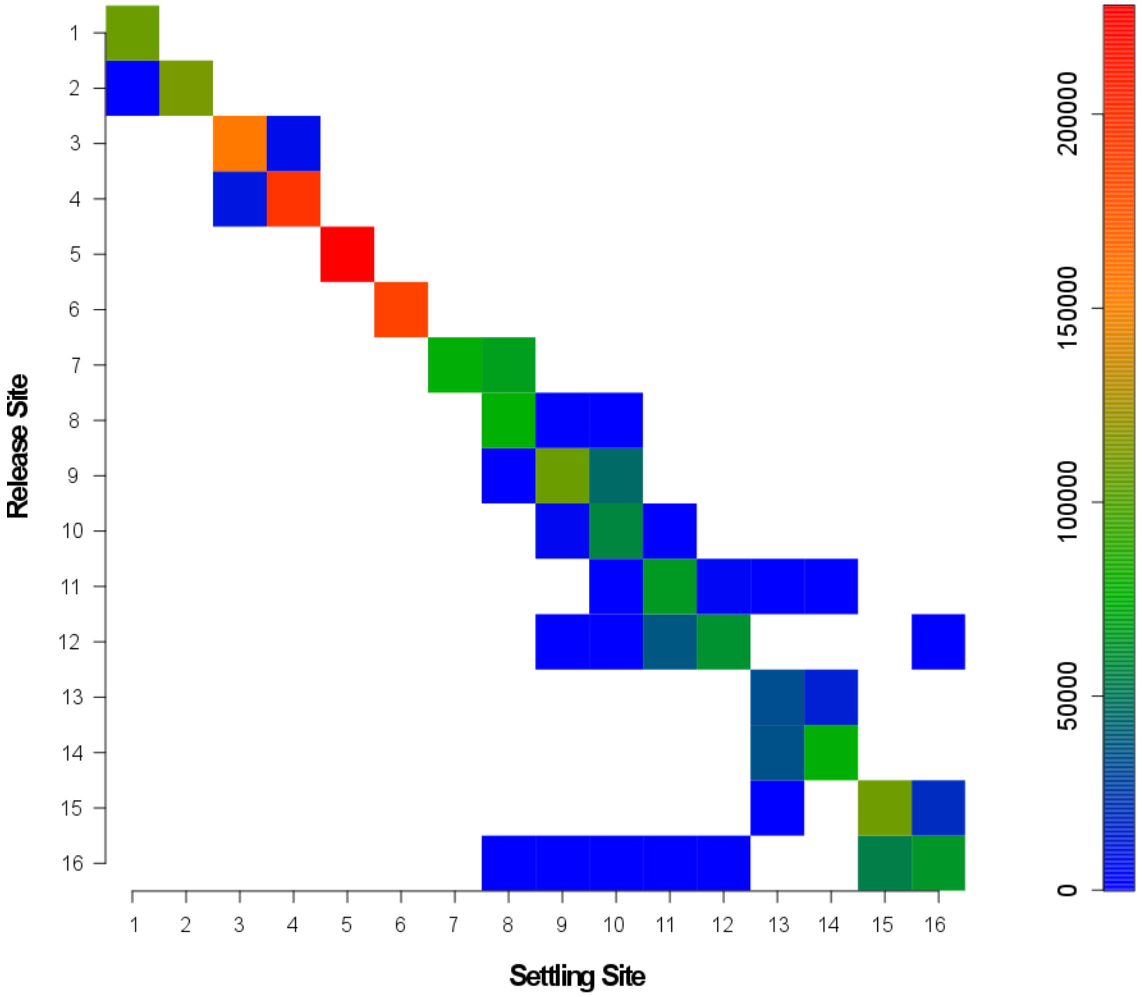

Figure S2. Simulated migration between pairs of sites. The present figure for migration rates of the planktonic larvae of the limpet *Siphonaria diemenensis* was based on oceanographic connectivity simulations (Model 1 for 1 generation).

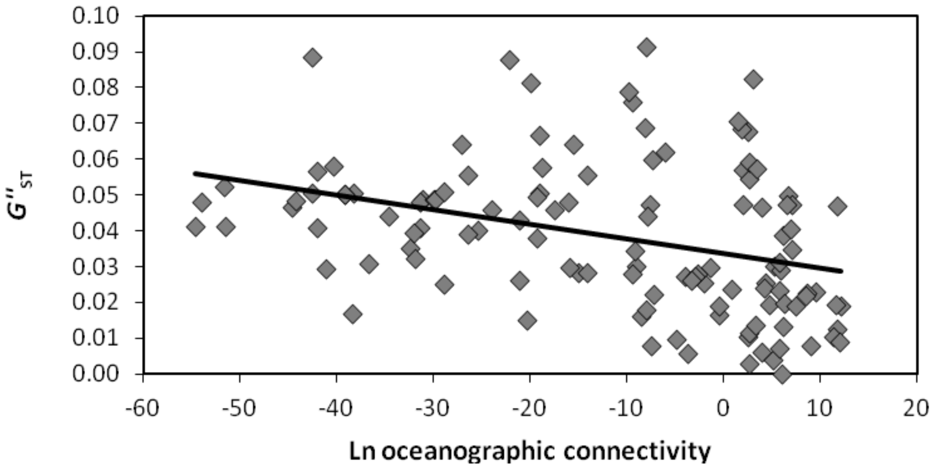

Figure S3. Regression plot depicting connectivity against genetic structure. The natural logarithm of connectivity from Model 2 for 5 generations was plotted against the genetic structure statistic G''_{ST}.

Supplementary Animations

Animation

Please see the following link for an animation of the ‘long’ model: Teske_animation.avi. The map in the animation was created using MATLAB2015b (http://uk.mathworks.com/products/matlab/).
